# Deterministic and probabilistic regularities underlying risky choices are acquired in a changing decision context

**DOI:** 10.1038/s41598-023-27642-z

**Published:** 2023-01-20

**Authors:** Andrea Kóbor, Eszter Tóth-Fáber, Zsófia Kardos, Ádám Takács, Noémi Éltető, Karolina Janacsek, Valéria Csépe, Dezso Nemeth

**Affiliations:** 1 Brain Imaging Centre, Research Centre for Natural Sciences, Magyar tudósok körútja 2, 1117 Budapest, Hungary; 2 Doctoral School of Psychology, ELTE Eötvös Loránd University, Izabella utca 46, 1064 Budapest, Hungary; 3 Institute of Psychology, ELTE Eötvös Loránd University, Izabella utca 46, 1064 Budapest, Hungary; 4 Brain, Memory and Language Research Group, Institute of Cognitive Neuroscience and Psychology, Research Centre for Natural Sciences, Magyar tudósok körútja 2, 1117 Budapest, Hungary; 5 Department of Cognitive Science, Budapest University of Technology and Economics, Egry József utca 1, 1111 Budapest, Hungary; 6 Cognitive Neurophysiology, Department of Child and Adolescent Psychiatry, Faculty of Medicine, TU Dresden, Fetscherstraße 74, 01307 Dresden, Germany; 7 Max Planck Institute for Biological Cybernetics, Max-Planck-Ring 8, 72076 Tübingen, Germany; 8 Centre of Thinking and Learning, Institute for Lifecourse Development, School of Human Sciences, Faculty of Education, Health and Human Sciences, University of Greenwich, Old Royal Naval College, Park Row, 150 Dreadnought, SE10 9LS London, UK; 9 Faculty of Modern Philology and Social Sciences, University of Pannonia, Egyetem utca 10, 8200 Veszprém, Hungary; 10 Université Claude Bernard Lyon 1, CNRS, INSERM, Centre de Recherche en Neurosciences de Lyon CRNL U1028 UMR5292, Bâtiment 462 – Neurocampus 95 Boulevard Pinel, F-69500 Bron, France

**Keywords:** Decision, Problem solving, Learning and memory

## Abstract

Predictions supporting risky decisions could become unreliable when outcome probabilities temporarily change, making adaptation more challenging. Therefore, this study investigated whether sensitivity to the temporal structure in outcome probabilities can develop and remain persistent in a changing decision environment. In a variant of the Balloon Analogue Risk Task with 90 balloons, outcomes (rewards or balloon bursts) were predictable in the task’s first and final 30 balloons and unpredictable in the middle 30 balloons. The temporal regularity underlying the predictable outcomes differed across three experimental conditions. In the deterministic condition, a repeating three-element sequence dictated the maximum number of pumps before a balloon burst. In the probabilistic condition, a single probabilistic regularity ensured that burst probability increased as a function of pumps. In the hybrid condition, a repeating sequence of three different probabilistic regularities increased burst probabilities. In every condition, the regularity was absent in the middle 30 balloons. Participants were not informed about the presence or absence of the regularity. Sensitivity to both the deterministic and hybrid regularities emerged and influenced risk taking. Unpredictable outcomes of the middle phase did not deteriorate this sensitivity. In conclusion, humans can adapt their risky choices in a changing decision environment by exploiting the statistical structure that controls how the environment changes.

## Introduction

Humans’ daily decisions mostly rely on experiences gathered under uncertain circumstances. For instance, dining out in a completely unknown restaurant might result in either a nice evening with lovely food or an unpleasant experience. In these circumstances, the probability distribution of the possible outcomes is unknown, but after repeated attempts, it can be more precisely estimated^1–3^. However, outcome probabilities can unexpectedly change or become completely unpredictable, undermining the predictions based on previous experiences^4,5^. Still, the already acquired knowledge of outcome probabilities might remain robust, depending on the deep structure of these probabilities and the perceived level of uncertainty^4,6–9^. Therefore, the present study aims to reveal whether representations of structurally different outcome probabilities are formed and remain stable if outcome probabilities unexpectedly change during risky decision making.

A large portion of previous research on decision making has attempted to understand the differences between decisions from description and decisions from experience. In the descriptive paradigms, outcome probabilities are *provided* a priori and known by the individuals; in the experiential paradigms, the probability distribution of the outcomes is *learned* from trial-by-trial experience because of repeated choices^1,2,10^. Confirming this distinction, different sets of behavioral tendencies have been observed within the two types of decisions (e.g.,^11–14^). These behavioral tendencies are assumed to be supported by different processes and accounted for by separate theories^2,3,10^. Meanwhile, successful modeling attempts have already connected the two decision domains and eventuated a better understanding of the underlying choice behavior (e.g.,^10,15–17^). Since, in day-to-day life, we make decisions mostly from experience in uncertain situations where the role of feedback is crucial, we used an experiential paradigm in this study.

To investigate the acquisition of outcome probabilities in a dynamically changing decision context with feedback, we implement experimental manipulations into the Balloon Analogue Risk Task (BART). In its original form, the BART measures real-life risk taking by simulating an uncertain decision environment with probabilistic reward and loss outcomes^18–22^. Task completion involves repeated decisions on either to inflate a virtual balloon larger and run the risk of a balloon burst or to collect the already accumulated reward because of previous successful balloon inflations.

One aspect of task performance is the acquisition or learning of outcome probabilities. This develops by experiencing successful balloon inflations and balloon bursts resulting from the adjustments of pumping behavior. As a measure of learning, some studies have quantified trial-by-trial reactivity in the BART (e.g.,^23–27^). Moreover, current computational models have become increasingly successful in capturing the learning aspect of task performance^28,29^. However, with experimental methods, it has scarcely been investigated how the direct manipulation of outcome probabilities alters the learning process and thereby risk-taking behavior in the BART^30–34^. These efforts are summarized next.

Some of the earlier studies using experimental manipulations investigated how initial experience with lucky (bursts after several pumps) or unlucky (bursts after a few pumps) series of balloons changed risk-taking behavior later in the task when burst probabilities became unbiased^30–32^. According to the results, individuals smoothly adjusted their risk-taking behavior to the changed probabilities. Meanwhile, this adjustment was more modest after unlucky initial events, suggesting persistent risk aversion. Other studies manipulated burst probabilities over several balloons^33,34^. The different burst probabilities were signaled with different colors of the balloons (cf.^19^), but participants were not informed about the exact burst probabilities related to each color. The order of colors was either randomized within each task block^34^ or consistent across a set of balloons and changed at the end of each set^33^. Mapping of colors to burst probabilities was either stable^34^ or changing over the task^33^. In the former case, participants had to learn the different burst probabilities, while in the latter case, they had to continuously update the learned probabilities in each set of balloons. According to the results, sensitivity emerged to the different burst probabilities, irrespective of whether the color mapping was stable or changing, as well as carryover effects were found across the burst probabilities^33^. To examine learning effects in the BART, the present study also applies different and changing burst probabilities, albeit in an unsignaled manner.

The deep structure of burst probabilities can be based on several types of regularities. Learning differently structured regularities has been extensively investigated in unsupervised learning environments, especially in the statistical-sequence learning literature^35–38^. In these environments, sensitivity to at least two types of sequential regularities—deterministic and probabilistic—has been found. In deterministic regularities, elements usually follow a fixed sequence. For instance, in a reaction time task, the location of each visual stimulus on the screen would follow the four-element sequence of “left, up, down, right” that repeats multiple times in a task block. Thus, consecutive sequence elements—the location of the next stimulus in the given example—can be predicted from the previous ones with 100% certainty. That is, “down” is always followed by “right” and “right” is always followed by “left”.

In contrast, if some noise (e.g., random elements) is embedded within the sequence, the resulting regularity becomes probabilistic. In the example above, the repeating sequence can be modified by inserting a random location out of the four possible ones between each of the two successive elements (i.e., left, random, up, random, down, random, right, random). Therefore, in probabilistic regularities, the predictability of a given element is less than 100%^35,39,40^. Altogether, while sure predictions can be formed with deterministic regularities, uncertain predictions arise with probabilistic regularities^41^. Deterministic and probabilistic information might not be treated along a continuum, since their learning has been modeled by two distinct hypothesis spaces^41,42^, and risky decisions based on probabilistic and deterministic regularities have been supported by different prefrontal areas in a complex gambling task^43^.

Consequently, manipulating the predictability of outcomes in the BART with the help of deterministic and probabilistic regularities could be a method to alter the learning process. We follow this approach in the present study. In the context of the BART, the deterministic and probabilistic aspects of the regularity pertain to the probability of balloon bursts. Controlled by the deterministic regularity, balloons burst with certainty after fixed pump numbers. This fixed sequence of pump numbers is repeated throughout the task, creating a dependency across balloons. With the probabilistic regularity, the risk of a balloon burst increases with each successive pump, but a balloon burst is not a sure event until a fixed point. The latter are the features of the original BART^19^, which can also be labeled as the “probabilistic” task version.

The attempt to respond to repeating regularities (similarity-based learning) has been shown to account for the behavioral tendencies observed during experiential decisions^5^. According to the “contingent average and trend” model and its extension, when making decisions, humans are assumed to be sensitive mostly to the sequential pattern of outcomes but sometimes also to the local trend of outcomes (most recent experiences)^5,44^. Thus, not all past experiences are considered as relevant, only the sequence of those outcomes that is similar to the most recent sequence. In other words, the similarity of current and previous events is judged according to sequences, and individuals expect the reappearance of these sequences^5^. Therefore, with the use of repeating deterministic and probabilistic sequences controlling balloon bursts, we could test whether individuals learn repeating regularities in the BART.

By alternating different probabilistic regularities according to a fixed repeating pattern, the combination of deterministic and probabilistic aspects can be attained. This “hybrid” manipulation could make predictions even more uncertain, since it changes the within-balloon uncertainty of burst probabilities over the task^4,41^. Therefore, this manipulation is also used in the present study. Uncertainty of predictions can be further manipulated by introducing transitions across different regularities. Earlier studies investigated how individuals track the transitions between random and non-random (regular) sequences of sensory stimuli^41,45^. As suggested by the phasic pupil dilatation responses of Zhao et al.^45^, the regular to random transition has been represented as an abrupt change in the stimulus regularities, signaling unexpected uncertainty. Meanwhile, without actively monitoring the transition, the opposite, random to regular transition has induced a gradual update or refinement of representations. This usually occurs under expected uncertainty and depends on evidence accumulation^4,45,46^. When the random to regular transition was investigated with the consideration of the type of the regular sequence, deterministic regularities were detected abruptly, while probabilistic regularities were detected gradually^41^.

Relying on these studies, we insert two between-balloons change points in the BART: We use a regular sequence over the first and final thirds of the task while random burst probabilities over the middle third. One third of the task (i.e., task phase) consists of 30 balloons. Thus, by reintroducing the regular sequence in the final phase, we use regular to random as well as random to regular transitions. This design enables us to test whether the representations of predictable regularities emerged over the first third of the task would remain persistent and become reactivated when the regularities reappear (cf.^6,47^). Hence, we examine whether the gradual effect of the random to regular transition alters due to the sensitivity to the reappearing regularity^4^. With both deterministic and probabilistic regularities, the differential effect of transitions as a function of uncertainty is also explored^41^.

**Figure 1:**
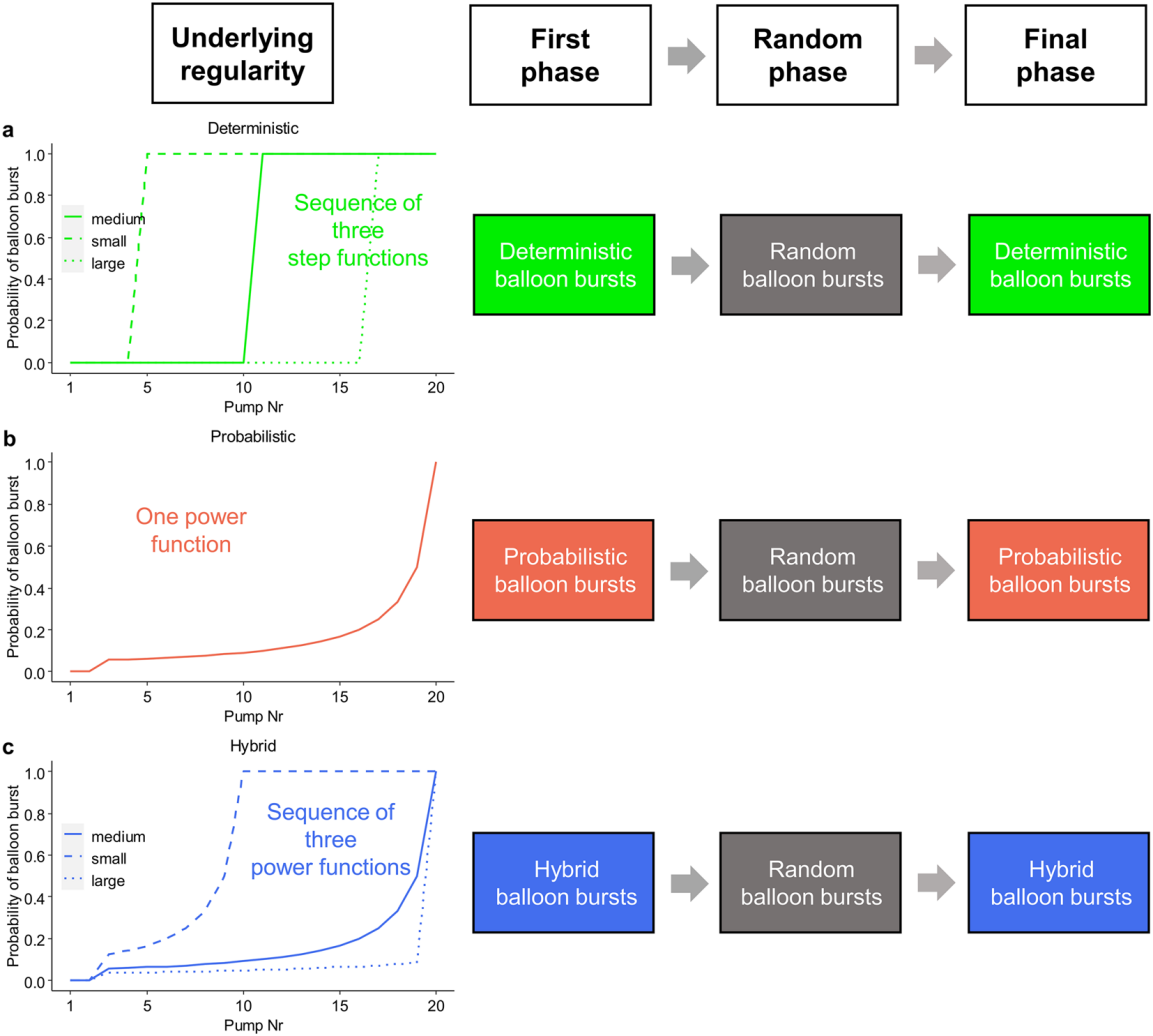
Design of the experiment. In this variant of the BART, two aspects of the reward scheme were manipulated. First, while decision outcomes—rewards or balloon bursts—were predictable in the first and final phases of the task, these were random (unpredictable) in the middle phase. Second, the structure of the regularity controlling the outcomes was manipulated. (**a**) In the deterministic condition, decision outcomes of every three balloons were controlled by a repeating sequence of three step functions. This ensured that the first balloon of the sequence could be inflated up to a medium size, the second to a small size, and the third one to a large size. Therefore, while these balloons burst with certainty after the 11th, 5th, and 17th pumps, respectively, burst probability was zero at lower pump numbers. (**b**) In the probabilistic condition, decision outcomes were controlled by a single truncated power function. While balloon bursts at the 1st and 2nd pumps were disabled, burst probability increased with each successive pump until the 20th where a balloon burst was certain. (**c**) In the hybrid condition, decision outcomes of every three balloons were controlled by a repeating sequence of three truncated power functions. This facilitated that the first balloon of the sequence could be potentially inflated up to a medium size, the second to a small size, and the third one to a large size. Balloon bursts at the 1st and 2nd pumps were disable and burst probability increased with each successive pump differently for the three balloon sizes. A balloon burst was certain at the 20th pump for medium and large balloons and at the 10th pump for small balloons. Each task phase consisted of 30 balloons. One participant performed only one experimental condition out of the three. Participants were not informed about the regularity controlling the outcomes in any of the conditions.

Altogether, the present experiment manipulated burst probabilities in the BART by creating three types of predictable regularities (see Fig. 1). One group of participants completed the original, probabilistic BART where larger balloons were coupled with increased risks of burst (see Fig. 1b). Another group completed a deterministic version where a three-balloon-long sequence was repeated. This sequence ensured balloons to burst always after medium, low, and high number of pumps, respectively (see Fig. 1a). These pump values were a priori fixed and remained the same. The last group completed a “hybrid” version where the medium, low, and high values of the three-balloon-long sequence changed in a probabilistic manner instead of being fixed (see Fig. 1c). The experimental design of all groups (conditions) consisted of three task phases: While the regularity was present in the first and final task phases (predictable phases), the regularity was completely removed for the middle phase (unpredictable phase). Thus, outcome probabilities became temporally random. Importantly, participants were not informed about either the presence or the absence of the regularity, and they did not have to track any change in the regularities. Nevertheless, they were asked in a post-task interview whether they became aware of the regularities. They completed the same amount of balloon trials (i.e., 30) in the first, middle, and final task phases.

We hypothesized that risk-taking behavior measured by the number of successful pumps would be higher in the predictable (first and final) phases than in the unpredictable (middle) phase, irrespective of condition (type of the underlying regularity). This was based on the concept that the acquisition of predictable sequences increases performance related to the elements of the sequence^35,37^. The number of pumps would increase mostly during the early trials of the BART, as experience with outcome probabilities accumulates and participants become more prone to take risks. This is a usually observed behavioral pattern in the original BART (e.g.,^22,48,49^), which we also expected with the present manipulations.

As stronger (or at least less uncertain) predictions can be formed with deterministic than with probabilistic regularities^41^, we hypothesized that sensitivity to the repeating balloon sequence would emerge in the deterministic condition. Furthermore, as the probabilistic regularities repeat in a deterministic fashion in the hybrid condition, we expected the emergence of sensitivity even to this structure. According to sequence-based similarity as a mechanism of acquiring outcome probabilities^5^, the sensitivity or learning effect would be captured by the different number of successful pumps on the respective balloon sizes (medium, small, large) and a more optimal risk-taking behavior. Knowledge of deterministic and probabilistic regularities has appeared as persistent and resistant to interference in unsupervised learning environments^6,47^. Therefore, the differentiation of balloon sizes and optimal risk-taking behavior would persist or become even more emphasized by the end of the task (in the final phase). However, the learning effect in the hybrid condition would emerge only in a gradual manner as evidence accumulates, because of the uncertainty generated by the different probabilistic regularities (cf.^4,45,46^). Based on the findings of Maheu, et al.^41^, we also hypothesized a gradual increase of pump number in the final phase of the probabilistic condition, after the random to regular transition occurred.

## Methods

### Participants

Altogether 141 healthy young adult participants were recruited from university courses. These undergraduate courses were explicitly dedicated for participation in different psychological experiments. Therefore, to fulfill the course requirements and receive course credit, students had to participate in some experiments during the semester. Beyond the partial course credit in exchange for participation in the present experiment, they were not given a bonus based on the total score gained in the task. However, according to the impressions of the experimenters during debriefing, they were still motivated to perform the task as if gains and losses were real. Participants were randomly assigned to three different experimental conditions labeled as deterministic (*n* = 46), probabilistic (*n* = 48), and hybrid (*n* = 47). These experimental conditions are described in detail below. One participant performed only one experimental condition to limit carryover effects across conditions. All participants had normal or corrected-to-normal vision and none of them reported a history of any neurological and/or psychiatric condition. None of them were excluded after participation. They provided written informed consent before enrollment. The experiment was approved by the United Ethical Review Committee for Research in Psychology (EPKEB) in Hungary and by the research ethics committee of Eötvös Loránd University, Budapest, Hungary; and it was conducted in accordance with the Declaration of Helsinki. Descriptive characteristics of participants are presented in Table 1.

**Table 1:** Descriptive data of demographic variables and BART performance in the three conditions.

Condition	Deterministic *M*(*SD*)	Probabilistic *M*(*SD*)	Hybrid *M*(*SD*)
*n*	46	48	47
Gender (Male/Female)	10/36	17/31	15/32
Age (years)	21.3 (1.6)	21.3 (2.2)	21.1 (1.8)
Education (years)	14.6 (1.4)	14.7 (2.0)	14.5 (1.6)
Mean pumps on non-burst balloons: first	7.2 (2.0)	7.7 (2.3)	6.6 (2.3)
Mean pumps on non-burst balloons: random	8.5 (2.1)	9.3 (2.6)	8.2 (2.5)
Mean pumps on non-burst balloons: final	9.3 (1.9)	9.2 (2.8)	8.2 (3.2)
Number of balloon bursts: first	9.1 (3.2)	10.9 (3.9)	11.3 (4.1)
Number of balloon bursts: random	13.1 (5.0)	13.2 (5.4)	12.1 (5.2)
Number of balloon bursts: final	9.0 (4.2)	14.1 (5.4)	14.6 (5.4)
Total score: first	715.7 (325.3)	663.7 (230.4)	500.0 (219.7)
Total score: random	702.3 (228.0)	777.2 (253.5)	686.2 (248.7)
Total score: final	1128.9 (423.4)	735.7 (261.4)	574.3 (263.6)

Each task phase consisted of 30 balloons. First and final phases had the same structure within conditions, but these phases differed across conditions (see main text for details). In the random (middle) phase, random balloon tolerance values were used, without the increasing burst probabilities within each balloon. The conditions did not differ in gender (*p* = 0.324), age (*p* = 0.852), and education (*p* = 0.757).

### Stimuli, task, and procedure

The detailed description of the task and procedure is provided in the Supplementary Methods; a summary can be read here. The surface structure and appearance of the BART were the same as described in previous studies^19,24,50–53^. Participants were instructed to achieve an as high score as possible by inflating empty virtual balloons on the screen without bursting them. They were also told that they were free to pump as much as they felt like, however, the balloon might burst. Each successful pump increased the size of the given balloon and the gained score by one point. After each successful pump, participants decided whether to continue inflating the balloon or to finish the given balloon trial by collecting the accumulated score. In the latter case, the balloon trial ended, and the accumulated score was transferred to a virtual permanent bank. An unsuccessful pump resulted in a balloon burst. This also ended the balloon trial and the accumulated score on the given balloon was lost, but this was not subtracted from the score in the permanent bank.

Participants had to inflate altogether 90 balloons that were assigned to three 30-balloon-long task phases. The first and final phases had the same deep structure *within* conditions, but they differed *across* the deterministic, probabilistic, and hybrid conditions. In the *deterministic* condition, a three-balloon-long sequence repeated 10 times in the first and final phases. The repeating sequence ensured balloon bursts to occur after fixed pump numbers (balloon tolerances). Thus, the first balloon of the sequence could be inflated up to a medium size, the second to a small size, and the third one to a large size (see Fig. 1a, Supplementary Table S1). In the *probabilistic* condition, the structure of the first and final phases followed that of the original task version^19^. Thus, each successive pump not only increased the chance to obtain a higher score but also the probability of a balloon burst and the accumulated score to be lost (see Fig. 1b, Supplementary Table S2). This contrasts with the deterministic condition where fixed balloon tolerances repeated. In the *hybrid* condition, again, a three-balloon-long sequence repeated 10 times in the first and final phases. However, instead of fixed balloon tolerances such as in the deterministic condition, three probabilistic regularities repeated to control balloon bursts. The three regularities facilitated the first balloon of the sequence to be potentially inflated up to a medium size, the second one to a small size, and the third one to a large size (see Fig. 1c, Supplementary Tables S2–S4). In the middle task phase of *all* conditions, tolerance values were *random* and burst probabilities did not increase within each balloon (across pumps). As these values were selected randomly for each balloon trial, the random “sequence” was not fixed across the conditions and participants.

Participants were told that they were going to inflate 30 balloons in each task phase at their own pace. They were also told that the starting score was zero in all phases; however, the overall total score at the end of the task was the sum of the total scores collected in each phase. The three phases were separated by short breaks in which participants could have had a few seconds to rest if needed. Importantly, participants were not informed about the regularity controlling the outcomes (balloon inflation or burst) in any of the conditions. Moreover, no information was provided about the change in this regularity across the phases, and they did not have to track this change. Therefore, this task measured decision making under uncertainty and experience-based risk, at least during the early trials^2,22,48,51,54^.

A short verbal interview with two questions was administered by the experimenters immediately after finishing the task to check whether participants gained awareness about the regularities guiding balloon bursts and/or the change in these regularities. The interview was recorded using the notebook’s built-in recording software. It was asked (1) how they solved the task, how they tried to maximize their scores; and (2) whether they noticed any regularity in the sequence of balloon bursts. The interviews were rated for two aspects. First, they were evaluated for the gained awareness about the regularities underlying balloon bursts in the deterministic and hybrid conditions. Second, they were rated for detecting the change in the underlying structure between the three phases of the task. Further details of the rating protocol are described in the Supplementary Note (entitled as Post-task interviews on the awareness of the hidden structure).

The experimental session took approximately one hour because other tasks measuring different aspects of cognitive performance (e.g., procedural learning, working memory) were also administered. Completion of the BART and the related verbal interview took 30–35 min. Results of the other tasks on a subsample of participants are reported in Zavecz et al.^55^.

### Data analysis

Data analysis was performed in three steps to evaluate in detail whether the hidden task structure influenced participants’ risk-taking behavior. Each step is described below in separate sections.

#### Analyzing the effects of outcome predictability and experience (Model 1)

The first analysis step tested the change of risk-taking behavior across the conditions as a function of *outcome predictability and experience* with the task. In this analysis, outcome predictability corresponded to task phase. Each balloon was assigned to either the first or the second half (i.e., 15 balloons) of the given task phase to track and interpret how experience with outcome probabilities changed risk-taking behavior as the task progressed.

The number of pumps on each balloon that did not burst was used as the dependent variable in the first analysis. The number of pumps on non-burst balloons (adjusted pump number) has been regarded as an index of deliberate, unbiased risk-taking behavior; and it is conventionally used in the BART literature^19,25,30,56,57^. In the current experimental context, pumps on non-burst balloons can indicate the true value of risk taking, which participants intentionally choose because of the acquired sensitivity to balloon tolerances.

We performed linear mixed-effects analysis. It is beneficial to analyze the current data this way, because, with these models, the non-independence of observations nested within participants and balloon trials (i.e., balloon pumps are repeated-measures observations) can be accounted for, the dependent variable does not have to be aggregated at the level of participants or balloon trials, and missing data are treated more suitably than in repeated-measures or mixed analyses of variance^58–60^.

The analysis was performed using the *lmer* function implemented in the *lme4* package^61^ of R^62^. The factors *Condition* (deterministic, hybrid, probabilistic), *Phase* (first, final), *Half* (1st, 2nd), and their *two-way* and *three-way* interactions were entered as fixed effects into the model. These factors were treated as categorical predictors. Participants were modeled as random effects (random intercepts). The model was fit with restricted maximum likelihood parameter estimates (*REML*). The *p*-values for fixed effects were computed using Satterthwaite’s degrees of freedom method with the *lmerTest* package^63^.

We used sum coding and the *probabilistic* condition, *first* phase, and *1st* half were chosen as the reference (“baseline”) levels of the given factors. Pair-wise comparisons were performed by the *emmeans* package^64^. Figures were created using the *ggplot2* package^65^. The schematic structure of the model is summarized below with the dependent variable on the left side of the “~” symbol and the predictor variables on the right side, (1 | participant) represents the random intercepts:

Model 1: Number of pumps on non-burst balloons in *all phases* ~ Condition, Phase, Half, Condition * Phase, Condition * Half, Phase * Half, Condition * Phase * Half + (1 | participant).

#### Analyzing the sensitivity to the repeating balloon sequence (Models 2 and 3)

The second analysis step *directly* tested whether participants in the deterministic and hybrid conditions *acquired sensitivity* to the repeating balloon sequence and *adjusted their risk-taking behavior accordingly.* This step involved data registered only in the first and final phases of the deterministic and hybrid conditions, where the repeating regularity defined the different balloon tolerances. Thus, the probabilistic condition and the random phases were omitted from this analysis. The deterministic and hybrid conditions were analyzed separately.

Again, the number of pumps on each balloon that did not burst was used as the dependent variable. The factors *Size* (medium, small, large), *Phase* (first, final), and their *two-way* interaction were entered as fixed effects into the separate linear mixed-effects models. The reference levels of the factors were *medium* balloon size and *first* phase. Otherwise, modeling was performed in the same way as in the case of Model 1. The schematic structures of Models 2 and 3 are summarized below:

Model 2: Number of pumps on non-burst balloons in the *first and final phases* of the *deterministic* condition ~ Size, Phase, Size * Phase + (1 | participant).

Model 3: Number of pumps on non-burst balloons in the *first and final phases* of the *hybrid* condition ~ Size, Phase, Size * Phase + (1 | participant).

#### Analyzing the sensitivity to the optimal pump number (Model 4)

As there was no repeating regularity in the probabilistic condition, previous models could not compare acquired sensitivity to the task’s structure across the conditions. Therefore, the third analysis step tested whether *sensitivity to the optimal pump number* in the predictable phases of the task differed across the conditions. As described in Supplementary Tables S2–S4, in the hybrid condition, 13, 6, and 19 could be considered as the optimal pump numbers for the medium, small, and large balloons, respectively. Similarly, 13 could be the optimal pump number in the probabilistic condition (see Supplementary Table S2). In the deterministic condition, the fixed balloon tolerance values (medium—10 pumps, small—4 pumps, large—16 pumps) were considered as the optimal pump numbers, because the probability of a balloon burst was zero until reaching the tolerance value, but it was one for the next pump (see Fig. 1a, Supplementary Table S1).

As the optimal pump number is interpretable only in the non-random phases, this analysis involved data registered only in the first and final phases. To determine how close a participant approached the optimum on each balloon, an efficiency score was calculated as the difference between the optimal and actual pumps numbers (optimal minus actual) divided by the optimal pump number. While a positive signed value of this ratio score indicates that pump number remained below the optimum, a negative signed value reflects that the pump number exceeded the optimum. *Lower* absolute values mean less deviation from the optimum; thus, *more efficient* (more optimal) pumping behavior. Because of using a ratio score, sensitivity to the optimum could be more easily compared across the conditions.

Different from the previous analyses, the dependent variable (efficiency score) was calculated based on the number of pumps on *all balloons*, including burst and non-burst balloons, as well. This measure could be more suitable for calculating an overall efficiency score, as it also contains those balloon trials in which participant could have intended to pump the balloons up to a larger, more optimal size but the balloons “accidentally” burst (i.e., in the probabilistic and hybrid conditions).

Regarding the analysis, the factors *Condition* (deterministic, hybrid, probabilistic), *Phase* (first, final), and their *two-way* interaction were entered as fixed effects into the linear mixed-effects model. The reference levels of the factors were *probabilistic* condition and *first* phase. Otherwise, modeling was performed in the same way as in the case of previous models. The schematic structure of Model 4 is summarized below:

Model 4: Efficiency score on all balloons in the *first and final phases* ~ Condition, Phase, Condition * Phase + (1 | participant).

## Results

To ease meta-analytic work, Table 1 shows the phase-wise performance (descriptive data) of the different conditions measured by classical indices of the BART such as the mean pumps on non-burst balloons, the number of balloon bursts, and the total score^25^. In the results section below, “pump number” refers to the number of pumps on non-burst balloons. The summaries of all effects included in the linear mixed-effects models (Models 1–4) are presented in Tables 2, 3 and 4. Only simplified statistics are provided in the main text. Since the post-task interviews were conducted and evaluated according to an unstandardized protocol and 12 of them were missing or inadequate, only the related descriptive results are provided below and in the Supplementary Note.

**Table 2:** Summary of the linear mixed-effects model testing the effects of outcome predictability and experience (Model 1).

Fixed effects	Estimate	*SE*	d﻿f	*t*-value	*p﻿*-value
(Intercept)	8.16	0.18	136.6	45.55	< 0.001
Deterministic	0.09	0.25	136.1	0.35	0.724
**Hybrid**	** − 0.53**	**0.25**	**136.9**	** − 2.11**	**0.037**
**Random phase**	**0.41**	**0.05**	**7468**	**9.06**	** < 0.001**
**Final phase**	**0.59**	**0.05**	**7469**	**12.95**	** < 0.001**
**2nd half**	**0.29**	**0.03**	**7467**	**9.19**	** < 0.001**
Deterministic * random phase	** − 0.24**	**0.06**	**7469**	** − 3.73**	** < 0.001**
Hybrid * random phase	**0.15**	**0.06**	**7467**	**2.38**	**0.018**
Deterministic * final phase	**0.30**	**0.06**	**7470**	**4.72**	** < 0.001**
Hybrid * final phase	** − 0.18**	**0.07**	**7468**	** − 2.76**	**0.006**
Deterministic * 2nd half	0.01	0.04	7467	0.25	0.806
Hybrid * 2nd half	0.00	0.05	7467	0.00	0.999
Random phase * 2nd half	** − 0.22**	**0.05**	**7466**	** − 4.89**	** < 0.001**
Final phase * 2nd half	** − 0.29**	**0.05**	**7466**	** − 6.37**	** < 0.001**
Deterministic * random phase * 2nd half	** − 0.14**	**0.06**	**7466**	** − 2.14**	**0.032**
Hybrid * random phase * 2nd half	0.09	0.06	7466	1.43	0.152
Deterministic * final phase * 2nd half	** − **0.04	0.06	7466	** − **0.56	0.574
Hybrid * final phase * 2nd half	0.05	0.07	7466	0.75	0.453
*Random effects*	Variance				
[Participants](Intercept)	4.38				

Dependent variable: Number of pumps on non-burst balloons. Coding scheme: sum coding. The reference levels of the factors were probabilistic condition, first phase, and 1st half. Significant effects are in **bold** (except the intercept). *SE*: standard error.

**Table 3:** Summary of linear mixed-effects models testing the sensitivity to the different balloon tolerances (balloon sizes) in the deterministic and hybrid conditions (Models 2–3).

Fixed effects	Estimate	*SE*	df	*t*-value	*p*-value
*Model 2* Deterministic					
(Intercept)	7.16	0.28	45.98	26.00	< 0.001
**Small**	** − 3.56**	**0.10**	**1879.99**	** − 35.22**	** < 0.001**
**Large**	**2.90**	**0.08**	**1877.47**	**37.48**	** < 0.001**
**Final phase**	**0.75**	**0.06**	**1877.02**	**12.31**	** < 0.001**
Small * final phase	** − 1.02**	**0.10**	**1875.93**	** − 10.23**	** < 0.001**
Large * final phase	**0.79**	**0.08**	**1875.27**	**10.27**	** < 0.001**
*Random effects*	Variance				
[Participants](Intercept)	3.32				

Fixed effects	Estimate	*SE*	df	*t*-value	*p*-value
*Model 3* Hybrid					
(Intercept)	7.04	0.34	46.29	20.58	< 0.001
**Small**	** − 0.90**	**0.12**	**1548.72**	** − 7.42**	** < 0.001**
**Large**	**0.53**	**0.09**	**1548.25**	**5.89**	** < 0.001**
**Final phase**	**0.61**	**0.07**	**1546.38**	**8.48**	** < 0.001**
Small * final phase	** − **0.18	0.12	1546.18	** − **1.53	0.127
Large * final phase	0.12	0.09	1545.43	1.37	0.170
*Random effects*	Variance				
[Participants](Intercept)	5.23				

Dependent variable in both models: Number of pumps on non-burst balloons. Coding scheme: sum coding. The reference levels of the factors were medium balloon size and first phase. Significant effects are in **bold** (except the intercept). *SE*: standard error.

**Table 4:** Summary of the linear mixed-effects model testing the sensitivity to the optimal pump number in the predictable task phases (Model 4).

Fixed effects	Estimate	*SE*	df	*t*-value	*p*-value
(Intercept)	0.318	0.010	138	32.65	< 0.001
**Deterministic**	** − 0.195**	**0.014**	**138**	** − 14.09**	** < 0.001**
**Hybrid**	**0.096**	**0.014**	**138**	**6.98**	** < 0.001**
**Final phase**	** − 0.041**	**0.003**	**8306**	** − 13.51**	** < 0.001**
Deterministic * final phase	** − 0.020**	**0.004**	**8306**	** − 4.53**	** < 0.001**
Hybrid * final phase	**0.012**	**0.004**	**8306**	**2.80**	**0.005**
*Random effects*	Variance				
[Participants](Intercept)	0.012				

Dependent variable: efficiency score (the difference of optimal minus actual pump number divided by the optimal pump number). This is determined for all balloons (irrespective of balloon burst). Coding scheme: sum coding. The reference levels of the factors were probabilistic condition and first phase. Significant effects are in **bold** (except the intercept). *SE*: standard error.

### The effects of outcome predictability and experience on risk-taking behavior (Model 1)

Model 1 tested the change of risk-taking behavior across the conditions as a function of outcome predictability and experience with the task. According to the results (see Table 2), pump number was significantly lower in the hybrid condition than the grand mean (β =  − 0.53, *t* =  − 2.11, *p* = 0.037). Furthermore, pump number was significantly higher in the random (β = 0.41, *t* = 9.06, *p* < 0.001) and final (β = 0.59, *t* = 12.95, *p* < 0.001) phases of the task, as well as in the 2nd half of the task phase (β = 0.29, *t* = 9.19, *p* < 0.001).

**Figure 2:**
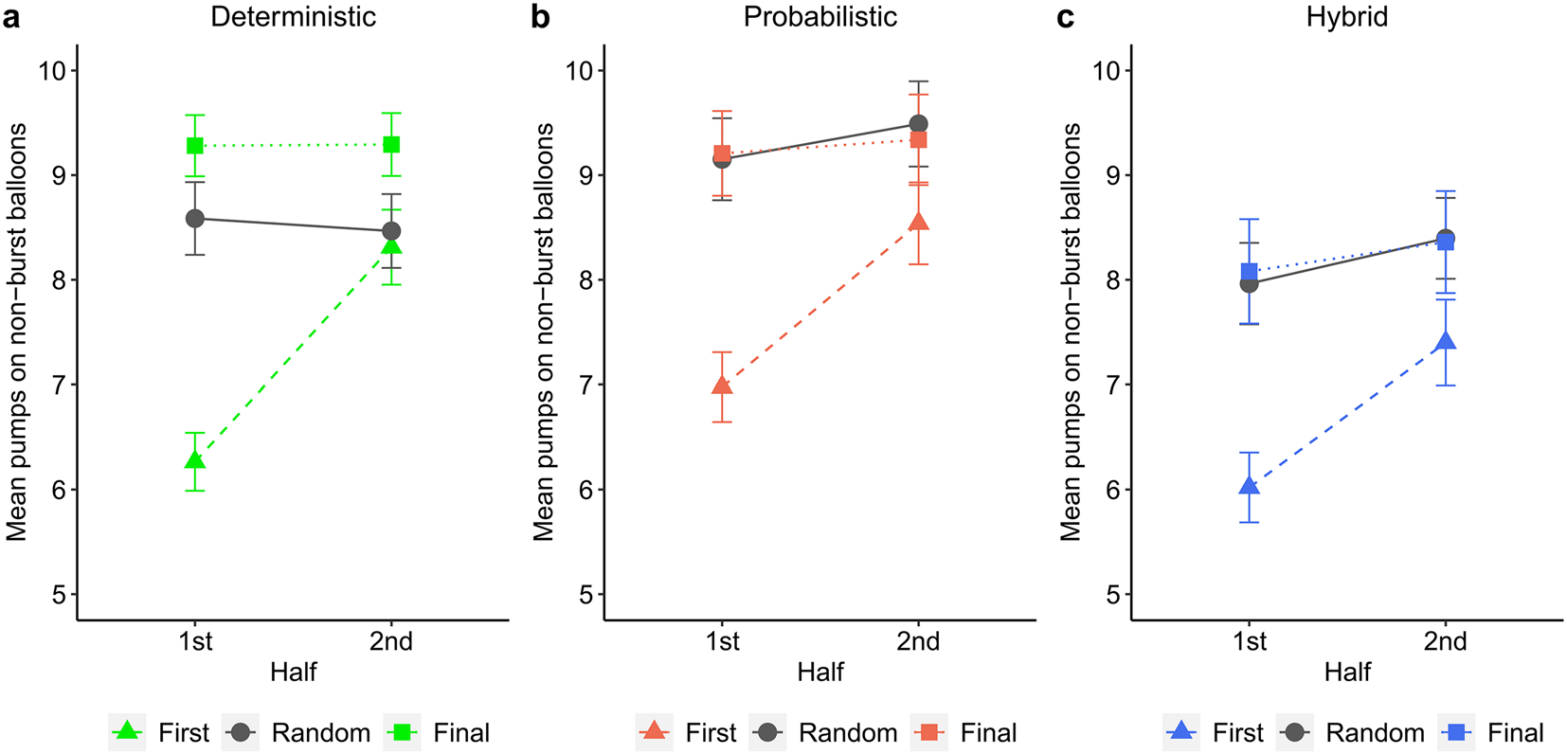
The effects of predictability and experience on risk taking. Group means of the mean number of pumps on non-burst balloons calculated for the 1st (balloons 1–15) and 2nd (balloons 16–30) halves of each phase (first, random, final) are presented separately in each condition (deterministic, probabilistic, hybrid). Error bars denote standard error of mean. Considering the 1st task half, pump number significantly increased across the first, random, and final phases in the deterministic (*p*s ≤ 0.001) but not in the other conditions (*p*s ≥ 0.855 between the random and final phases). Considering the 2nd task half, pump number was similar in the first and random phases (*p* = 0.575) and higher in the final phase (*p*s < 0.001) in the deterministic condition. Meanwhile, pump number was comparably higher in the random and final phases than in the first phase in the hybrid and probabilistic conditions (*p*s ≤ 0.002).

However, as shown by the significant Random Phase * 2nd Half (β =  − 0.22, *t* =  − 4.89, *p* < 0.001) and Final Phase * 2nd Half (β =  − 0.29, *t* =  − 6.37, *p* < 0.001) interactions and pair-wise comparisons, pump number increased significantly between the task halves only in the first phase (*p* < 0.001) and not in the random and final phases (*p*s ≥ 0.208). Thus, the steep rise of pump number characterized only the first task phase (see Fig. 2). Meanwhile, although outcomes were unpredictable (random) in the middle phase, this did not decrease overall pump number.

More importantly, the significant two-way interactions of Deterministic * Random Phase, Hybrid * Random Phase, Deterministic * Final Phase, and Hybrid * Final Phase (all *p*s ≤ 0.018; see Table 2) indicate that in the deterministic condition, pump number increased significantly from phase to phase, even from the random to the final phase (*p* < 0.001, see Fig. 2a). However, this final vs. random increase was not present in the hybrid and probabilistic conditions (*p*s ≥ 0.509, see Fig. 2b, c). In addition, although pump number abruptly increased from the first to the random phase in the hybrid condition, in the final phase, it was still lower than in the deterministic (*p* = 0.042) and probabilistic conditions (*p* = 0.055).

The three-way interaction of Deterministic * Random Phase * 2nd Half was also significant (β =  − 0.14, *t* =  − 2.14, *p* = 0.032). This suggests that the increase of pump number over the task phases differing across the conditions was modulated by task halves. These differences across the task halves are detailed in the caption of Fig. 2. In essence, the 2nd half of the random phase was comparable to that of the first phase in the deterministic condition, while it was comparable to that of the final phases in the other two conditions (see Fig. 2). This pattern of results emerged because the random phase as compared with the predictable phases was differently performed in the deterministic than in the probabilistic and hybrid conditions.

Altogether, the deterministic condition was characterized by a large increase in pumps between the first and final phases and comparable pump numbers between the 2nd half of the first phase and the entire random phase. This suggests that the regular to random transition might have influenced risk taking differently in the deterministic condition than in the others. When outcomes became predictable again in the final phase, only participants of the deterministic condition increased risk taking as compared with the random phase. However, these analyses do not show whether the repeating balloon sequence was indeed acquired and reactivated after the random to regular transition. The second set of analyses investigates this question.

### Sensitivity to the repeating balloon sequence

#### Deterministic condition (Model 2)

Model 2 tested whether participants of the deterministic condition acquired sensitivity to the repeating balloon sequence and adjusted their risk-taking behavior to fit this sequence. Results showed (see Table 3) that they differentiated across the three balloon sizes: Small balloons were pumped significantly less (β =  − 3.56, *t* =  − 35.22, *p* < 0.001) and large balloons were pumped significantly more (β = 2.90, *t* = 37.48, *p* < 0.001) than the grand mean and the medium balloons (*p*s < 0.001). Furthermore, balloons were pumped to a significantly larger size in the final than in the first task phase (β = 0.75, *t* = 12.31, *p* < 0.001).

As per the significant Small * Final Phase (β =  − 1.02, *t* =  − 10.23, *p* < 0.001) and Large * Final Phase (β = 0.79, *t* = 10.27, *p* < 0.001) interactions, the difference between the balloon sizes increased by the final phase (see also Table 3). While both medium (*p* < 0.001) and large (*p* < 0.001) balloons were pumped to a significantly larger size, small balloons were pumped to a similar extent during the final task phase (see Supplementary Table S5). This suggests that knowledge of the repeating sequence became more consistent with experience and guided deliberate risk-taking behavior.

**Figure 3:**
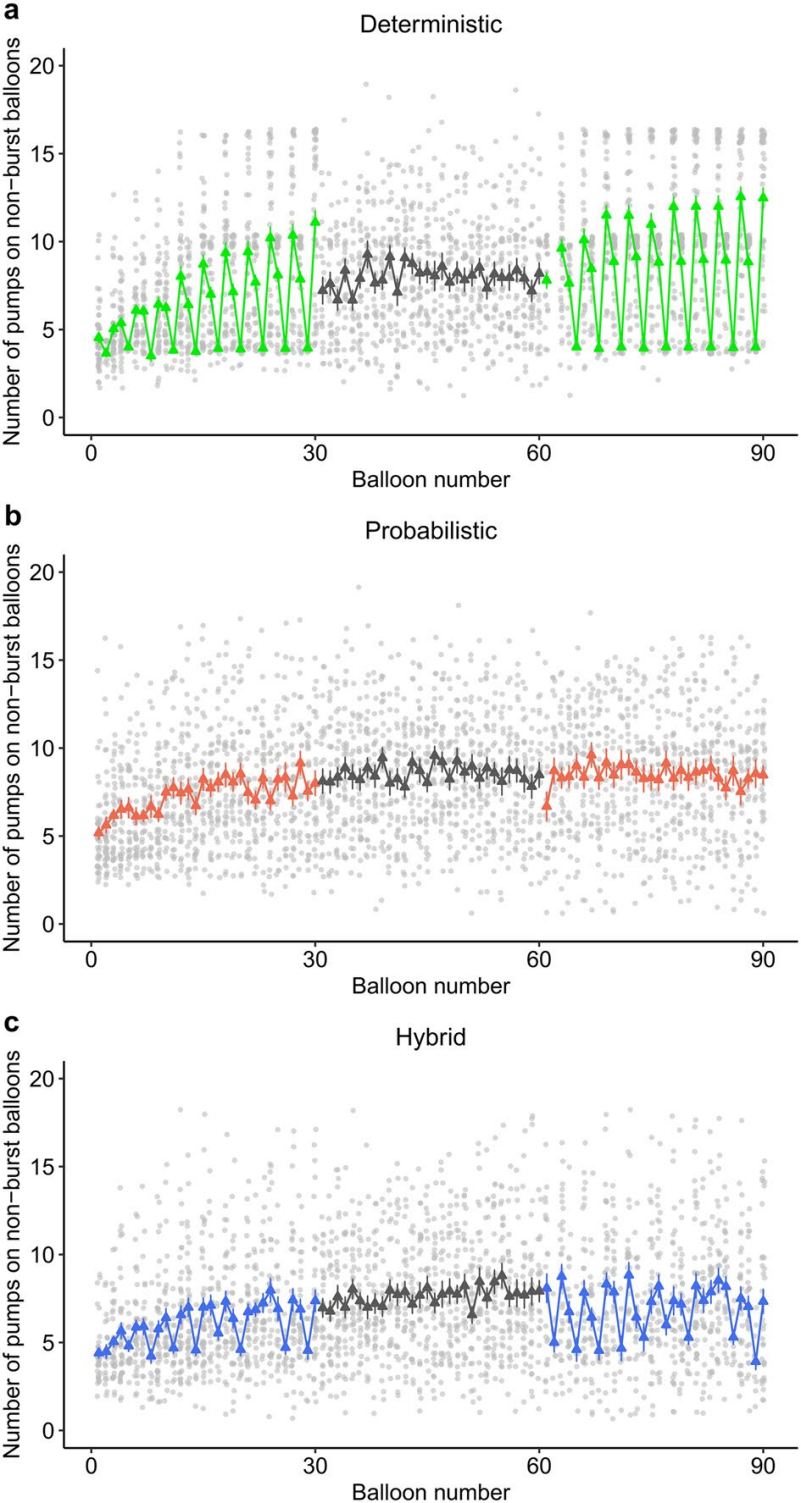
Sensitivity to the underlying regularities. The number of pumps on non-burst balloons are presented across all balloons of the task separately in each condition (deterministic, probabilistic, hybrid). Triangles denote the group (condition) mean of pumps on each balloon, light grey circles denote individual data points jittered to prevent overlap, and error bars denote standard error of mean in all figure parts. Each phase of the task encompasses 30 balloons (first phase: balloon numbers 1–30, random phase: balloon numbers 31–60, final phase: balloon numbers 61–90). Means and standard errors of the random phase are depicted in grey in all figure parts. Means and standard errors of the first and final manipulated phases are depicted in green (deterministic), coral (probabilistic), and blue (hybrid), respectively. Discontinuities in the lines connecting mean values indicate missing data for certain balloons in all figure parts (i.e., all participants of the given condition burst the particular balloon).

These results are also visible in Fig. 3a: In the first phase of the deterministic condition, pump numbers start to gradually follow the varying balloon tolerances of the medium-small-large repeating sequence. In the final phase, this pattern appears to become stable. Altogether, participants of the deterministic condition adjusted their risk-taking behavior to fit the balloon tolerances and differentiated across the three tolerance values.

#### Hybrid condition (Model 3)

Model 3 tested whether participants of the hybrid condition acquired sensitivity to the repeating balloon sequence. Results showed (see Table 3) that small balloons were pumped significantly less than the grand mean (β =  − 0.90, *t* =  − 7.42, *p* < 0.001) and the medium balloons (*p* < 0.001). Although large balloons were pumped significantly more than the grand mean (β = 0.53, *t* = 5.89, *p* < 0.001), their pump numbers did not differ significantly from that of medium balloons (*p* = 0.449). Altogether, participants differentiated between small and medium balloons but did not between medium and large balloons. Regarding the task phase, significantly more pumps occurred in the final than in the first phase (β = 0.61, *t* = 8.48, *p* < 0.001). The Small * Final Phase and Large * Final Phase interactions were non-significant (see Table 3, Supplementary Table S5), indicating that any knowledge of the repeating sequence did not reliably strengthen by the final task phase.

In sum, participants of the hybrid condition partially adjusted their risk-taking behavior to fit the repeating probabilistic regularities. It seems that they remained insensitive to the difference between medium and large balloons even by the final task phase (see also Fig. 3c). It is likely that participants acquired that “small” and “larger” balloons followed one another instead of fully tracking the medium-small-large repeating sequence.

#### Post-task interviews

Post-task interviews tested whether participants became aware of the hidden task structure. Therefore, these interview ratings could complement the behavioral findings on acquired sensitivity. In line with the behavioral results, post-task interviews (see Supplementary Note) suggest that while most participants (72.5%) reported explicit awareness (partial or full) of the repeating balloon sequence in the deterministic condition, only a few participants (19%) reported (partial) awareness in the hybrid condition. Furthermore, 60% as opposed to 16.7% explicitly reported a change between task phases in the deterministic vs. the hybrid condition; and, in the latter condition, no participant at all reported the actual change.

### Sensitivity to the optimal pump number (Model 4)

Model 4 tested whether sensitivity to the optimal pump number in the predictable first and final phases of the task differed across the conditions. According to the results (see Table 4), while the deterministic condition showed significantly less deviation from the optimal pump values (β =  − 0.195, *t* =  − 14.09, *p* < 0.001), the hybrid condition showed significantly more (β = 0.096, *t* = 6.98, *p* < 0.001), as compared with the grand mean. Furthermore, pumping behavior was significantly more optimal in the deterministic condition than in the hybrid and probabilistic ones (*p*s < 0.001), while the latter two conditions did not differ from one another (*p* = 0.993; deterministic *M* = 0.123; hybrid *M* = 0.414; probabilistic *M* = 0.417).

Pumping behavior was significantly more optimal in the final than in the first phase (β =  − 0.041, *t* =  − 13.51, *p* < 0.001; first *M* = 0.359; final *M* = 0.277). As per the significant Deterministic * Final Phase (β =  − 0.020, *t* =  − 4.53, *p* < 0.001) and Hybrid * Final Phase (β = 0.012, *t* = 2.80, *p* = 0.005) interactions, the optimization (change) of pumping behavior from the first to the final phase occurred to a larger extent in the deterministic condition and to a lesser extent in the hybrid one (deterministic *M* = 0.121; hybrid *M* = 0.058; probabilistic *M* = 0.067).

Altogether, pumping behavior was the most optimal in the deterministic condition, which became more emphasized by the final task phase. Participants of the hybrid and probabilistic conditions showed comparable but less optimal pumping behavior, which strengthened by the final task phase to a lesser extent than in the deterministic condition.

## Discussion

### Summary of findings

This study investigated how the transitions between predictable and unpredictable outcomes and the deep structure of predictable outcomes influenced risk-taking behavior in an experience-based risky decision-making environment. To this end, while outcomes were predictable in the first and final phases of the BART, these were unpredictable in the middle phase. The deep structure of predictable outcomes was also manipulated. Either a repeating balloon sequence with deterministic and probabilistic regularities or a single probabilistic regularity was present in one of the three experimental conditions. Participants were not informed about these regularities and that the transitions between task phases denoted a change in the predictability of outcomes.

Risk taking in the probabilistic and hybrid conditions increased in the first predictable phase and remained consistent in the remainder of the task, as usually observed in the original BART. This suggests a rapidly emerging general sensitivity to the predictable outcomes. When the predictable outcomes reappeared in the final phase, risk taking increased only in the deterministic condition. In this condition, specific sensitivity to the repeating balloon sequence also emerged, as shown by the successful differentiation of medium, small, and large balloons, which became more emphasized in the final phase. Most participants gained explicit knowledge of the deterministic regularity. This was also reflected by their risk-taking behavior approaching the optimal level, especially in the final task phase. In the hybrid condition, the specific sensitivity to the repeating balloon sequence was partial, as shown by the differentiation of small and “larger” balloons. This sensitivity did not change in the final phase. Knowledge of the hybrid regularity remained largely implicit. In line with these results, participants of the hybrid condition showed less optimal risk-taking behavior, which was comparable to that of the probabilistic condition. In the deterministic and hybrid conditions, unpredictable outcomes did not seem to influence risk taking, at least in terms of the number of successful pumps in the final phase. Relatedly, risk taking in the probabilistic condition was found to be insensitive to changes in predictability across the task phases.

### Sensitivity to the underlying regularities

Earlier work used color cues to indicate the different burst probabilities in the BART and found sensitivity to these probabilities^33,34^. Importantly, the present study unveiled sensitivity even without signaling any characteristic of the underlying probabilities, at least when these followed a repeating pattern. This is in line with those studies that manipulated the early balloons in an unsignaled manner and found the adjustment of risk taking later in the task^30–32^.

The present results also suggest that the acquired knowledge of deterministic and probabilistic regularities is robust and resistant to interference triggered by the interposed unpredictable outcomes. Persistent knowledge of deterministic and probabilistic regularities has been observed in unsupervised learning environments, as well^6,47^. The learning of both deterministic and probabilistic regularities could occur implicitly or explicitly^35,66–68^. In the present experiment, while knowledge of the deterministic regularity was mostly explicit, knowledge of the hybrid regularity remained mostly implicit.

Regarding the acquired knowledge, participants of the hybrid condition did not differentiate between large and medium balloons, as opposed to those in the deterministic condition. Since we used probabilistic regularities in the hybrid condition, bursts of both medium and large balloons could have been experienced even after comparable pump numbers. These negative events might have obscured the differentiation of medium and large balloons. Considering all task phases in the hybrid condition, outcomes varied highly. High variance of outcomes coupled with negative events could have resulted in decreased risk taking, which hindered the full exploration of the hybrid structure (cf.^14,26,69^).

Note that because of a programming error, the outcomes of the 23rd balloons in the first and final phases of the hybrid condition were controlled by the probabilistic regularity of the medium balloons instead of the small ones. Thus, the 8th repetition (22nd, 23rd, 24th balloons) of the medium-small-large balloon sequence was violated in both phases, since a medium-medium-large sequence appeared instead. After excluding the 22nd, 23rd, and 24th balloons from the first and final phases, the Size by Phase model was refit to the data of the hybrid condition. Results nearly identical to the original ones were obtained, as summarized in Supplementary Table S6. In addition, it seems that the pattern of successful pumps followed the repeating sequence at its next repetition right after the sequence violation (see in Fig. 3c). Altogether, the partial sensitivity to the repeating balloon sequence in the hybrid condition remained robust even if the sequence was violated.

### Interpretation of results

In the deterministic condition, there were no reliable signals other than the repeating sequence that predicted balloon bursts. Therefore, in line with the similarity-based model of Plonsky et al.^5^, participants could follow the repeating sequence when comparing current and past experiences. When deciding on whether to pump the given balloon further, they might have recognized that current outcomes matched a particular sequence of the recalled past outcomes. As the reappearance of the repeating sequence was possibly expected, experiences gathered during the unpredictable phase could have been labeled as “irrelevant” and did not deteriorate the sequence-based decision strategy in the final phase of the deterministic condition. This interpretation of the observed risk-taking behavior would be in line with the notion that sensitivity to sequential regularities and the detection or search of environmental patterns, even they are not present (e.g.,^70,71^), could be fundamental aspects of learning^35,44^. In addition, this similarity-based learning model can provide a common ground for the interpretation of various phenomena derived from the research fields of experience-based decision making and unsupervised statistical-sequence learning^38,44^.

In the hybrid condition, the uncertainty of predictions was higher because of the probabilistic nature of the repeating sequence^41^. Thus, similarity functions other than sequence-based similarity and additional behavioral strategies could have been used when making decisions. Further research should attempt to clarify the processes underlying choice behavior observed in the hybrid condition. The combined reinforcement learning diffusion decision model (RLDDM) might be a good candidate for this purpose, because it could predict the interaction of choice preferences and response times to capture learning effects^15,72^. Analysis of response times in the BART—the time needed to initiate the next pump on a given balloon—could reveal the perception of elevated risk levels and uncertainty^58^. Hence, in future studies, response times can indicate sensitivity to changes in the underlying regularities.

Since the deep structure of the task was unknown in advance, explorative behavior characterized all conditions in the first phase, reflected by the rapid increase of successful pumps. In the deterministic condition, the regular to random transition induced fundamental changes in the underlying regularity and resulted in (possibly) unexpected outcomes^46^. This might explain why risk taking in the random phase stabilized at the level achieved by second half of the first phase. In the hybrid condition, however, it is not unequivocal whether this transition resulted in unexpected or expected outcomes. Although the use of different probabilistic regularities in the repeating sequence increases uncertainty, some sensitivity to the repeating regularity still emerged until this point (i.e., the differentiation of balloon sizes). Thus, even the first transition might have violated outcome expectations, but this cannot be clearly inferred from the current results. The second, random to regular transition resulted in abrupt behavior adjustment in both the deterministic and hybrid conditions to fit the repeating balloon sequence (cf.^41^). The probabilistic regularities in the hybrid condition did not delay this behavior adjustment. The abrupt change in both conditions might be due to the task relevance of the random to regular transition^45^.

Particularly, although participants were not instructed to identify the underlying regularities, based on the post-task interviews, many of them were motivated to do so even in the unpredictable phase to achieve better performance on the task. This would be in line with sequence-based similarity as the process underlying choice behavior. Therefore, if they continuously built representations of the underlying regularities, the random to regular transition signaled the violation of expectations. This unexpected change in the underlying regularities could have promoted further explorative behavior^4,46^, which facilitated matching the current outcomes with the previously experienced repeating sequence of outcomes. However, after recognizing the stability and separability of outcomes in the final phase, participants of the deterministic condition could have started to exploit their reactivated knowledge of the repeating sequence. This was not that clear in the hybrid condition, as risk taking in the final phase was lower than in the deterministic condition.

Only cautious assumptions can be formed about how participants of the probabilistic condition processed the task structure. This condition could be characterized by expected uncertainty^4^, due to the structural similarities of the predictable and unpredictable phases. Particularly, balloon tolerance values varied between two and 19 in all task phases. Although burst probabilities did not increase with each pump in the unpredictable phase, the risk level still increased: Individuals had to consider the possibility to gain even more reward (the added points increased with each pump) or to lose the already accumulated reward^27,73^. Therefore, burst probabilities might have been perceived comparably across the task phases. This would be in accordance with the consistent pump number seen from the unpredictable phase throughout the remaining balloons (cf. Fig. 3b) and the subjective reports (post-task interviews). These reports suggest that the structure was “unchanged” throughout the task in the probabilistic condition (see Supplementary Note).

### Limitations and directions for future research

Some limitations of the present work should be noted. First, burst probabilities differed across the conditions because of how we constructed the regularities, which should be avoided in future designs. Second, it has been found that individuals with internalizing symptoms, such as in anxiety and depression, might show difficulties in the adaptation to uncertain decision-making environments because of altered learning rates^34,74–76^. Therefore, it would be interesting to investigate whether and how, for instance, trait anxiety at the subclinical level and decision making are related when different and changing underlying regularities are applied in the BART.

Third, most of our participants were females because we recruited them from undergraduate courses that were also characterized by similar gender distributions. Previous studies found lower risk taking in females than in males and other gender differences when performing the BART (e.g.,^19,34,77,78^). Although the general level of risk taking can be different between the genders, in the present study, the full (deterministic condition) and partial (hybrid condition) sensitivity to the repeating balloon sequence emerged in both the male and female subsamples, similarly to the whole sample (analyses are not reported). Still, future work should strive for equal gender distribution to ensure that the observed effects of risk taking can be generalized to the entire population. Fourth, separating task phases with short breaks might have involuntarily signaled the introduction of new rules, although we intended to use unsignaled and unexpected transitions. The positions of breaks could have directly helped participants to search for the repeating sequence or part of the sequence in the final phase, contributing to the observed persistency of knowledge. A novel experimental design should present balloon trials in a single phase with short breaks that do not fall on the boundaries of the different contexts.

Fifth, participants were not paid a bonus based on task performance because of the lack of resources. However, recent work has implied that not only the behavioral indices but also the electrophysiological correlates of negative feedback processing are enhanced if real money instead of hypothetical reward is used in the BART^79,80^. It would be interesting to see whether the degree of learning changes across the experimental conditions because of a paid bonus. Sixth, the number of pumps involving every balloon trial (both burst and non-burst balloons) could have been used in the first analysis. This index would increase the reliability of the data, because trials of those participants who inflate the balloons to a larger size and thereby more likely experience balloon bursts are also considered^25^. Nevertheless, results of the first analysis (Model 1) would have been similar even with this dependent variable (see Supplementary Table S7).

Finally, computational models that capture differences in learning as a function of the regularities’ deep structure and the type of transition might be considered^15,28,57^, complemented by tracking the electrophysiological correlates of uncertainty and feedback processing^4,58^. Computational modeling would be particularly helpful to directly test the current interpretations of the findings (described in earlier sections) derived from existing theoretical approaches and previous experimental and modeling work. With a computational approach, the parameter differences across the conditions could be compared to investigate behavior adjustment.

### Conclusions

This study showed that sensitivity to repeating regularities underlying the outcomes of risky decisions can emerge even if the regularities are temporarily missing. This sensitivity develops without informing individuals about the presence, absence, and reappearance of the regularities. The emergence of this sensitivity depends mostly on the type of the regularities: While completely predictable deterministic regularities can be acquired easily, uncertain predictions based on probabilistic regularities are more challenging. Experiencing intermittent unpredictable outcomes does not seem to disrupt the acquired representations because of their resistance to interfering information. In sum, by the acquisition and expectation of sequential patterns, the present results suggest fast and robust adaptation to changing outcome probabilities in experience-based risky decision making. Moreover, the results also highlight how unsupervised and reward-based learning of structures can be linked.

## Supplementary Information

Supplementary Information.

## Data Availability

Data are available in the following online repository: https://osf.io/bwzfa/?view_only=f0920ce7759144ceb4be81318cc76f75.
