# Supplementary material for: Deterministic and probabilistic regularities underlying risky choices are acquired in a changing decision context
Source: Sci Rep. 2023 Jan 20;13:1127. doi: 10.1038/s41598-023-27642-z (PMC9859780; doi:10.1038/s41598-023-27642-z)
Supplement: Supplementary file 1 — Supplementary Information. [file 41598_2023_27642_MOESM1_ESM.pdf]

Supplementary Information of the manuscript entitled

"Deterministic and probabilistic regularities underlying risky choices are acquired in a changing decision context"

Authors: Andrea Kóbor, Eszter Tóth-Fáber, Zsófia Kardos, Ádám Takács, Noémi Éltető, Karolina Janacsek, Valéria Csépe, Dezso Nemeth

Supplementary Tables

Table S1. Task structure in the deterministic condition.

Balloon	First	Random	Final
1	10	11	10
2	4	8	4
3	16	7	16
4	10	5	10
5	4	2	4
6	16	8	16
7	10	12	10
8	4	2	4
9	16	8	16
10	10	16	10
11	4	5	4
12	16	11	16
13	10	16	10
14	4	6	4
15	16	18	16
16	10	10	10
17	4	2	4
18	16	12	16
19	10	17	10
20	4	8	4
21	16	10	16
22	10	18	10
23	4	14	4
24	16	6	16
25	10	17	10
26	4	12	4
27	16	9	16
28	10	16	10
29	4	8	4
30	16	11	16

Note. Numbers in *italics* in the column labelled as “Balloon” denote the sequential numbers of balloons. Balloon tolerance values in first and final phases were fixed, followed a medium-small-large (10-4-16) sequence that repeated ten times, and were identical across participants. Balloon tolerance values were random and varied across participants in the random phase; an example for a series of random values is presented here. Balloon tolerance value means that if a participant inflated the given balloon one pump further, the balloon burst.

Table S2. Characteristics of the probabilistic regularity controlling balloon tolerances in the first and final phases of the probabilistic condition and medium balloon tolerances in the hybrid condition.

Pump Nr	p burst	p inflation	Reward	Accumulated score	Potential loss	Potential gain	Gain–loss difference
1	0	1	1	0	0	1	1
2	0	1	2	1	0	2	2
3	0.056	0.944	3	3	0.17	2.83	2.67
4	0.059	0.941	4	6	0.35	3.76	3.41
5	0.063	0.938	5	10	0.63	4.69	4.06
6	0.067	0.933	6	15	1.00	5.60	4.60
7	0.071	0.929	7	21	1.50	6.50	5.00
8	0.077	0.923	8	28	2.15	7.38	5.23
9	0.083	0.917	9	36	3.00	8.25	5.25
10	0.091	0.909	10	45	4.09	9.09	5.00
11	0.100	0.900	11	55	5.50	9.90	4.40
12	0.111	0.889	12	66	7.33	10.67	3.33
13	0.125	0.875	13	78	9.75	11.38	1.63
14	0.143	0.857	14	91	13.00	12.00	**-1.00**
15	0.167	0.833	15	105	17.50	12.50	**-5.00**
16	0.200	0.800	16	120	24.00	12.80	**-11.20**
17	0.250	0.750	17	136	34.00	12.75	**-21.25**
18	0.333	0.667	18	153	51.00	12.00	**-39.00**
19	0.500	0.500	19	171	85.50	9.50	**-76.00**
20	1	0	0	190	190	0	**-190**

Note. The probability of balloon bursts (“p burst”) and successful balloon inflations (“p inflation”) are denoted for each possible pump (“Pump Nr”). Balloon bursts for the first and second pumps are disabled, and the probability of balloon burst is one for the 20th pump, when the accumulated score on the given balloon is lost. Each successful pump increases “Reward” by one point. “Potential loss” is the product of the probability of balloon burst (“p burst”) and the *already* “Accumulated score” before the current pump. “Potential gain” is the product of the probability of balloon inflation (“p inflation”) and “Reward” for the current pump. The expected value of inflating the balloon (“Gain–loss difference”) is positive until the 13th pump. Thus, taking a further risk by additional pumps is advantageous until this point, which is considered as the optimal pump number. After, the expected value is negative (**boldfaced** values); therefore, additional pumps are disadvantageous. This

probabilistic regularity controlled all balloons of the probabilistic condition in first and final phases and the medium balloons of the hybrid condition in first and final phases.

Table S3. Characteristics of the probabilistic regularity controlling small balloon tolerances in the first and final phases of the hybrid condition.

Pump Nr	p burst	p inflation	Reward	Accumulated score	Potential loss	Potential gain	Gain–loss difference
1	0	1	1	0	0	1	1
2	0	1	2	1	0	2	2
3	0.125	0.875	3	3	0.38	2.63	2.25
4	0.143	0.857	4	6	0.86	3.43	2.57
5	0.167	0.833	5	10	1.67	4.17	2.50
6	0.200	0.800	6	15	3.00	4.80	1.80
7	0.250	0.750	7	21	5.25	5.25	**0.00**
8	0.333	0.667	8	28	9.33	5.33	**-4.00**
9	0.500	0.500	9	36	18.00	4.50	**-13.50**
10	1	0	0	45	45	0	**-45**

Note. The probability of balloon bursts ("p burst") and successful balloon inflations ("p inflation") are denoted for each possible pump ("Pump Nr"). Balloon bursts for the first and second pumps are disabled, and the probability of balloon burst is one for the 10th pump, when the accumulated score on the given balloon is lost. Each successful pump increases "Reward" by one point. "Potential loss" is the product of the probability of balloon burst ("p burst") the *already* "Accumulated score" before the current pump. "Potential gain" is the product of the probability of balloon inflation ("p inflation") and "Reward" for the current pump. The expected value of inflating the balloon ("Gain–loss difference") is positive until the 6th pump. Thus, taking a further risk by additional pumps is advantageous until this point, which is considered as the optimal pump number. After, the expected value is zero or negative (**boldfaced** values); therefore, additional pumps are disadvantageous. This probabilistic regularity controlled the small balloons of the hybrid condition in first and final phases.

Table S4. Characteristics of the probabilistic regularity controlling large balloon tolerances in the first and final phases of the hybrid condition.

Pump Nr	p burst	p inflation	Reward	Accumulated score	Potential loss	Potential gain	Gain–loss difference
1	0	1	1	0	0	1	1
2	0	1	2	1	0	2	2
3	0.036	0.964	3	3	0.11	2.89	2.79
4	0.037	0.963	4	6	0.22	3.85	3.63
5	0.038	0.962	5	10	0.38	4.81	4.42
6	0.040	0.960	6	15	0.60	5.76	5.16
7	0.042	0.958	7	21	0.88	6.71	5.83
8	0.043	0.957	8	28	1.22	7.65	6.43
9	0.045	0.955	9	36	1.64	8.59	6.95
10	0.048	0.952	10	45	2.14	9.52	7.38
11	0.050	0.950	11	55	2.75	10.45	7.70
12	0.053	0.947	12	66	3.47	11.37	7.89
13	0.056	0.944	13	78	4.33	12.28	7.94
14	0.059	0.941	14	91	5.35	13.18	7.82
15	0.063	0.938	15	105	6.56	14.06	7.50
16	0.067	0.933	16	120	8.00	14.93	6.93
17	0.071	0.929	17	136	9.71	15.79	6.07
18	0.077	0.923	18	153	11.77	16.62	4.85
19	0.083	0.917	19	171	14.25	17.42	3.17
20	1	0	0	190	190	0	**-190**

Note. The probability of balloon bursts ("p burst") and successful balloon inflations ("p inflation") are denoted for each possible pump ("Pump Nr"). Balloon bursts for the first and second pumps are disabled, and the probability of balloon burst is one for the 20th pump, when the accumulated score on the given balloon is lost. Each successful pump increases "Reward" by one point. "Potential loss" is the product of the probability of balloon burst ("p burst") the *already* "Accumulated score" before the current pump. "Potential gain" is the product of the probability of balloon inflation ("p inflation") and "Reward" for the current pump. The expected value of inflating the balloon ("Gain–loss difference") is always positive until the 19th pump, which is considered as the optimal pump number. This probabilistic regularity controlled the large balloons of the hybrid condition in first and final phases.

Table S5. Mean pumps on non-burst balloons in the deterministic and hybrid conditions separately for the different balloon tolerances (balloon sizes) in the first and final phases. Estimated marginal means (upper table) and observed means (lower table) are presented.

	Phase		First	Final
Deterministic	Size	Medium	6.84	8.8
		Small	3.87	3.34
		Large	8.52	11.6
	Phase		First	Final
Hybrid	Size	Medium	6.73	8.08
		Small	5.71	6.56
		Large	6.84	8.3

	Phase		First	Final
Deterministic	Size	Medium	6.58	8.70
		Small	3.86	3.98
		Large	8.44	11.51
	Phase		First	Final
Hybrid	Size	Medium	6.37	7.42
		Small	4.68	4.85
		Large	6.66	7.99

Table S6. Summary of the linear mixed-effects model testing the sensitivity to the different balloon tolerances (balloon sizes) in the hybrid condition after omitting the 22nd, 23rd, and 24th balloons.

Fixed effects	Estimate	*SE*	df	*t*-value	*p*-value
(Intercept)	6.94	0.34	46.01	20.63	< .001
**Small**	**-0.85**	**0.12**	**1370.19**	**-6.90**	**< .001**
**Large**	**0.52**	**0.09**	**1369.22**	**5.55**	**< .001**
**Final Phase**	**0.62**	**0.07**	**1367.39**	**8.39**	**< .001**
Small * Final Phase	-0.22	0.12	1367.33	-1.76	.078
Large * Final Phase	0.12	0.09	1366.28	1.32	.188
Random effects	Variance				
[Participants](Intercept)	5.04				

Note. Dependent variable: Number of pumps on non-burst balloons. The model was fit to the data of the hybrid condition without the 22nd, 23rd, and 24th balloons of the first and final phases because of a programming error. Coding scheme: sum coding. The reference levels of the factors were medium balloon size and first phase. Significant effects are in **bold** (except the intercept). *SE*: standard error. The results and main conclusions of this analysis remained similar to that of the original one (Model 3, hybrid condition). Although small balloons were pumped less and large balloons were pumped more (*p*s < .001), participants differentiated only between small and medium balloons ($p < .001$) and not between large and medium balloons ($p = .388$). More pumps occurred in the final than in the first phase.

Table S7. Summary of the linear mixed-effects model testing the effects of outcome predictability and experience on the number of pumps including *all balloons* (variant of Model 1 with a different dependent variable).

Fixed effects	Estimate	*SE*	df	*t*-value	*p*-value
(Intercept)	7.47	0.13	138	58.87	< .001
Deterministic	0.31	0.18	138	1.74	.084
**Hybrid**	**-0.60**	**0.18**	**138**	**-3.35**	**.001**
**Random Phase**	**0.35**	**0.04**	**12520**	**9.05**	**< .001**
**Final Phase**	**0.36**	**0.04**	**12520**	**9.25**	**< .001**
**2nd Half**	**0.24**	**0.03**	**12520**	**8.80**	**< .001**
**Deterministic * Random Phase**	**-0.29**	**0.06**	**12520**	**-5.24**	**< .001**
**Hybrid * Random Phase**	**0.29**	**0.06**	**12520**	**5.28**	**< .001**
**Deterministic * Final Phase**	**0.41**	**0.06**	**12520**	**7.28**	**< .001**
**Hybrid * Final Phase**	**-0.30**	**0.06**	**12520**	**-5.45**	**< .001**
Deterministic * 2nd Half	0.04	0.04	12520	0.94	.346
Hybrid * 2nd Half	0.02	0.04	12520	0.41	.679
**Random Phase * 2nd Half**	**-0.21**	**0.04**	**12520**	**-5.42**	**< .001**
**Final Phase * 2nd Half**	**-0.20**	**0.04**	**12520**	**-5.20**	**< .001**
**Deterministic * Random Phase * 2nd Half**	**-0.20**	**0.06**	**12520**	**-3.68**	**< .001**
**Hybrid * Random Phase * 2nd Half**	**0.15**	**0.06**	**12520**	**2.77**	**.006**
Deterministic * Final Phase * 2nd Half	0.03	0.06	12520	0.62	.536
Hybrid * Final Phase * 2nd Half	0.00	0.06	12520	0.08	.938
Random effects	Variance				
[Participants](Intercept)	2.16				

Note. Dependent variable: Number of pumps on all balloons (both burst and non-burst balloons). Coding scheme: sum coding. The reference levels of the factors were probabilistic condition, first phase, and 1st half. Significant effects are in **bold** (except the intercept). *SE*: standard error. The results and main conclusions of this analysis remained similar to that of the original one with the non-burst pumps as the dependent variable (i.e., Model 1). Only subtle differences were found between the two analyses. In particular, the Hybrid * Random Phase * 2nd Half interaction became significant (cf. Table 2). Pair-wise comparisons revealed that pump number in the hybrid condition was significantly lower than in the other conditions in the first and final phases (*p*s ≤ .041). More importantly, a significant *decrease* of pump number from the random to the final phase in the hybrid condition was found ($p < .001$). This decrease was also present when considering the 1st ($p = .020$) and 2nd ($p < .001$) halves separately, and, because of this, the first vs. final difference was non-significant in the 2nd half ($p = .150$). The lack of first vs. final difference in the 2nd half was also true for the probabilistic condition ($p = .354$).

Supplementary Methods

Detailed description of the task and procedure

The surface structure and appearance of the BART were the same as described in previous studies [1-6]. The task was implemented in Presentation (v. 18.1, Neurobehavioral Systems). Participants were instructed to achieve an as high score as possible by inflating empty virtual balloons on the screen without bursting them. They were also told that they were free to pump as much as they felt like, however, the balloon might burst. Each successful pump increased the size of the given balloon and the gained score by one point. Therefore, participants could gain one point for the first successful pump, two more points for the second pump (i.e., the accumulated score for a given balloon was three), three more for the third (i.e., the accumulated score was six), and so on (see Supplementary Tables S1-S4). The accumulated score was persistently shown in the middle of the balloon.

After each successful pump, participants decided whether to continue inflating the balloon or to finish the given balloon trial by collecting the accumulated score. In the latter case, the balloon trial ended, and the accumulated score was transferred to a virtual permanent bank. The content of the permanent bank (total score) together with the score collected from the previous balloon also appeared constantly on the screen. An unsuccessful pump resulted in a balloon burst. This also ended the balloon trial and the accumulated score on the given balloon was lost, but this was not subtracted from the score in the permanent bank. After the end of a balloon trial, a feedback panel showing either the accumulated score or a picture of balloon burst was presented; then, the next trial started with a new empty balloon appearing on the screen. Two response keys of a keyboard were available either to pump the balloon or to collect the accumulated score.

The deep structure of the task was modified and differed across the deterministic, probabilistic, and hybrid conditions. Participants had to inflate altogether 90 balloons that

were assigned to three 30-balloon-long phases. The first and final phases had the same structure within conditions, but these phases differed across conditions. The middle phase had an identical structure across all conditions.

In the *deterministic* condition, a three-balloon-long sequence repeated 10 times in the first and final phases, adding up the altogether 30 balloons in both phases. In short, burst probabilities of every three balloons were controlled by a repeating sequence of three step functions (see Fig. 1a). In particular, we determined fixed values for the *maximum* number of successful pumps on each of the three balloons of the sequence (see Supplementary Table S1). These balloon tolerance values meant that the probability of a balloon burst was zero until reaching the tolerance value, but it was one for the next pump [7]. The tolerance values of the three balloons were 10, 4, and 16, respectively. Therefore, the first balloon of the sequence could be inflated up to a medium size, the second to a small size, and the third one to a large size. The score maximally gained on such a sequence was 55, 10, and 136 points, respectively. The tolerance values of the repeating medium-small-large sequence were identical across participants. In the middle phase with 30 balloons, tolerance values were random and varied across participants. For each balloon, an integer between two and 19 was randomly selected. Again, a successful balloon increase was assured if the pump number did not exceed the given tolerance value and a sure burst happened if participants inflated the given balloon one pump further than the tolerance value. We have chosen this interval because balloon burst was enabled only after the third pump, and the maximum number of successful pumps was 19 in those task versions that were used in our previous studies (e.g., [3,4,7]). Since a predetermined number of pumps resulted in either a balloon burst or a balloon increase for each balloon type of the repeating sequence, we labeled this condition as deterministic.

In the *probabilistic* condition, the structure of the first and final phases followed that of the original task version [1]. Thus, each successive pump not only increased the chance to obtain a higher score but also the probability of a balloon burst and the accumulated score to be lost (see Supplementary Table S2). This contrasts with the first and final phases of the deterministic condition and with the middle phase of all conditions with random tolerance values. The regularity determining balloon bursts in the first and final phases of the probabilistic condition followed three principles: (1) balloon bursts for the first and second pumps were disabled; (2) the maximum number of successful pumps for each balloon was 19; (3) the probability of a balloon burst was 1/18 for the third pump, 1/17 for the fourth pump, and so on for each further pump until the 20th, where the probability of a balloon burst was 1/1. In other words, burst probability for a given pump was defined by the following truncated power function: $p_{\text{burst}} = (\text{tolerance} + 2 - \text{pump}_n)^{-1}$, where pump_n is the nth pump ($n \geq 3$) on a given balloon and tolerance is equal to 19 (see Fig. 1b). In the middle phase, random balloon tolerance values between two and 19 were used, without the increasing burst probabilities within each balloon (across pumps).

In the *hybrid* condition, a three-balloon-long sequence repeated 10 times in the first and final phases. We labeled this condition as hybrid because three probabilistic regularities repeated in a deterministic manner. The three regularities facilitated the first balloon of the sequence to be potentially inflated up to a medium size, the second one to a small size, and the third one to a large size (see Supplementary Tables S2-S4). Particularly, after the first two pumps where balloon bursts were disabled, for the *small* balloons, the probability of a balloon burst was 1/8 for the third pump, 1/7 for the fourth pump, and so on for each further pump until the 10th, where the probability of a balloon burst was 1/1 (i.e., the maximum number of successful pumps for each balloon was nine). For the *medium* balloons, the same probabilistic regularity as in the case of the probabilistic condition was used with a maximum tolerance of

19 pumps. For the *large* balloons, the probability of a balloon burst was 1/28 for the third pump, 1/27 for the fourth pump, and so on for each further pump until the 20th, where the probability of a balloon burst was 1/1 (i.e., the maximum number of successful pumps for each balloon was 19). In short, burst probabilities of every three balloons were defined by a repeating sequence of three truncated power functions (see above) with tolerance values of 19 (medium), 9 (small), and 29 (large), respectively, with the restriction that burst probability was a priori fixed as 1 at the 20th pump for large balloons (see Fig. 1c).

As a result of the three types of probabilistic regularities, the *optimal* pump number differed across the balloon types: It was approximately 13, 6, and 19 for medium, small, and large balloons, respectively (for the calculation of the optimal pump number, see Supplementary Tables S2-S4). Note that although the maximum size to which large balloons could be inflated up was the same as for the medium balloons in the hybrid condition, burst probability increased as a function of pumps in a less abrupt manner (see Fig. 1c). Thus, burst probability for the 19th pump was 1/12 in the case of the large balloons while it was 1/2 in the case of the medium balloons. We decided to limit the maximum balloon tolerance in 19 pumps (190 points) to maintain the length of the task within a reasonable range and to equalize the subjective experience of balloon sizes across conditions. It should also be noted that because we used probabilistic regularities, balloon bursts could be experienced even after fewer pumps; in these cases, the medium and large balloons might not have been perceived as larger or different than the small balloons. In the middle phase of the hybrid condition, again, random balloon tolerance values between two and 19 were used, without the increasing burst probabilities within each balloon.

Participants were told that they were going to inflate 30 balloons in each task phase at their own pace. They were also told that the starting score was zero in all phases, however, the overall total score at the end of the task was the sum of the total scores collected in each

phase. The three phases were separated by short breaks in which participants could have had a few seconds to rest if needed and the experimenter started the program code that controlled the next phase. Importantly, participants were not informed about the regularity determining the outcomes (balloon inflation or burst) in any of the conditions. Moreover, no information was provided about the change in this regularity across the phases, and they did not have to track this change. The zero probability of balloon bursts after the first and second pumps was also unknown to them.

Supplementary Note

Post-task interviews on the awareness of the hidden structure

1. Rating protocol

The experimenters were not blind to the research question and the experimental condition of the participants. The interviews were rated by one of the authors (E.T-F.) who was not blind to the research question or to the experimental conditions either. Independent raters were not involved. The short verbal interviews were rated for two aspects. First, they were evaluated for the gained awareness about the regularities underlying balloon bursts in the deterministic and hybrid conditions. Second, they were rated for detecting the change in the underlying structure between the three phases of the task.

In the *deterministic* condition, we examined whether participants became aware of the repetition of the 10 – 4 – 16 tolerance values (or the repetition of 55 – 10 – 136 points). If participants reported any kind of regularity, their answers were further examined for whether they reported the *completely* correct sequence or only a *partly* correct sequence. Participants were classified into the completely correct sequence group even if they gained complete awareness of the sequence only in the final phase. In the case of partly correct sequence reports, two types of answers could be differentiated: (1) participants who noticed the three-balloon-long sequence but could not completely report the correct sequence and (2) participants who noticed only a part of the sequence (e.g., they noticed that the maximum number of successful pumps were four for every third balloon, but they did not detect any other regularity). Similarly, in the *hybrid* condition, we examined whether participants became aware of the repeating regularity. If a participant reported any kind of regularity, their interviews were further examined in the same manner for whether they noticed the three-balloon-long sequence or only a part of it. As no repeating sequence was present in the *probabilistic* condition, those interviews were not rated for this aspect.

The interviews were also rated for detecting the change in the underlying structure between the three phases of the task. If a participant reported *any change* in the balloon bursts between the phases, their answers were further examined to determine whether they detected the *correct difference* between the phases. For an answer to be classified as correct, gaining complete awareness of the sequence in the deterministic and hybrid conditions were not a requirement. Answers of participants who detected only part of the three-balloon-long sequence but reported that the first and final phases contained regularities whereas the middle one did not were marked as correct.

2. Results

Due to technical difficulties, four recordings were missing in the deterministic condition, five in the hybrid condition, and one in the probabilistic condition. Two additional recordings could not be evaluated in the deterministic condition due to inadequate questioning. In sum, 40 interviews were evaluated in the deterministic condition, 42 in the hybrid condition, and 47 in the probabilistic condition.

In the *deterministic* condition, 72.5% of the participants noticed any regularities during the task. Out of these 29 participants, ten participants (34.5%) reported the completely correct sequence, 11 participants (37.9%) noticed the presence of the three-balloon-long sequence but could not report the sequence correctly and, 8 participants (27.6%) noticed only part of the sequence. The remaining participants (n = 11, 27.5% of the whole sample) did not report anything about the regularities.

In the *hybrid* condition, none of the participants became completely aware of the repeating sequence. Eight participants (19.0%) noticed some regularities behind the balloon bursts, they reported part of the sequence (i.e., a balloon with low tolerance was regularly

followed by a balloon with high tolerance). 34 participants (81.0%) did not notice anything about the underlying regularities of the balloon bursts.

The interviews were also rated for whether participants gained awareness of the differences in the underlying regularities between the three phases. In the *deterministic* condition, 24 participants (60.0%) reported that they noticed a change in the balloon bursts between the phases. Out of the 24 participants, 13 of them (54.2%) reported the correct change (i.e., that the balloon bursts followed a sequence in the first and final phases but not in the middle phase) and 11 participants (45.8%) reported an incorrect difference. 16 participants (40.0%) did not observe any change between the phases. In the *hybrid* condition, 7 participants (16.7%) detected a change, but none of them provided the correct answer. In the *probabilistic* condition, 3 participants (6.4%) noticed a change but none of them correctly.

References

1 Lejuez, C. W. *et al.* Evaluation of a behavioral measure of risk taking: the Balloon Analogue Risk Task (BART). *J Exp Psychol Appl* **8**, 75-84, doi:10.1037//1076-898X.8.2.75 (2002).

2 Fein, G. & Chang, M. Smaller feedback ERN amplitudes during the BART are associated with a greater family history density of alcohol problems in treatment-naive alcoholics. *Drug Alcohol Depend* **92**, 141-148, doi:10.1016/j.drugalcdep.2007.07.017 (2008).

3 Kóbor, A. *et al.* Different strategies underlying uncertain decision making: Higher executive performance is associated with enhanced feedback-related negativity. *Psychophysiology* **52**, 367-377, doi:10.1111/psyp.12331 (2015).

4 Kardos, Z. *et al.* Age-related characteristics of risky decision-making and progressive expectation formation. *Behav Brain Res* **312**, 405-414, doi:10.1016/j.bbr.2016.07.003 (2016).

5 Takács, Á. *et al.* High trait anxiety is associated with attenuated feedback-related negativity in risky decision making. *Neurosci Lett* **600**, 188-192, doi:10.1016/j.neulet.2015.06.022 (2015).

6 Éltető, N. *et al.* Do adolescents take more risks? Not when facing a novel uncertain situation. *Cogn Dev* **50**, 105-117, doi:10.1016/j.cogdev.2019.03.002 (2019).

7 Kóbor, A. *et al.* Adaptation to recent outcomes attenuates the lasting effect of initial experience on risky decisions. *Sci Rep* **11**, 10132, doi:10.1038/s41598-021-89456-1 (2021).
